# 

**Published:** 1875-02

**Authors:** 


Vol. V. FEBRUARY, 1875. No. 2.

### THE SOUTHERN

### MEDICAL RECORD:

PUBLISHED MONTHLY AT

ATLANTA, GEORGIA:

LOCAL EDITORS:

THOS. S. POWELL, M.D.

W. T. GOLDSMITH, M.D. R. C. WORD, M.D.

ASSOCIATE EDITORS:

T. CURTIS SMITH, M.D., - - - - Middleport, Ohio.
SWAN M. BURNETT, M.D., - - - Knoxville, Tennessee.
ALBAN S PAYNE M.D., - - - - Markham's, Virginia.
A. R. KILPATRICK, M.D., - - - Navasota, Texas.
A. A. LYON, M.D., - - - - Columbus, Mississippi.
G. A. BAXTER, M.D., - - - Chattanooga, Tenn.
J. B. MATTISON, M.D., - - - - Chester, New Jersey.

*"QUICQUID PRÆCIPIES ESTO BREVIS."*

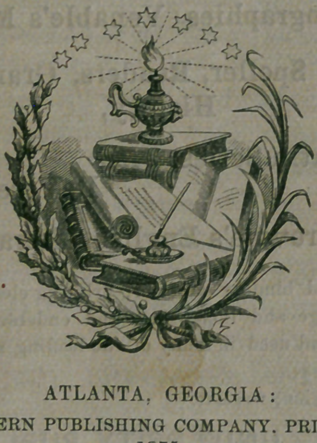

ATLANTA, GEORGIA:
SOUTHERN PUBLISHING COMPANY. PRINTERS.
1875.

Terms, $3 00 Per Annum, in Advance. Single Number, 35 Cents.

